# Overweight, obesity, and food consumption in Galapagos, Ecuador: a window on the world

**DOI:** 10.1186/s12992-018-0409-y

**Published:** 2018-09-12

**Authors:** Wilma B. Freire, William F. Waters, Diana Román, Elisa Jiménez, Estefania Burgos, Philippe Belmont

**Affiliations:** Institute for Research in Health and Nutrition, Universidad San Francisco de Quito, Diego de Robles y Pampite s/n, Cumbayá, Quito, Ecuador

**Keywords:** Diet, Overweight and obesity, Mixed methods, Ecuador

## Abstract

**Background:**

In order to understand why rates of overweight and obesity are so high in the Ecuadorian province of Galapagos, this study analyzes changes in household food expenditures and perceptions and practices related to food consumption patterns. Galapagos is understood as an unusual but not unique case because conditions there graphically illustrate trends observed in communities and countries worldwide. A mixed methods approach was employed: a quantitative component was based on expenditures for foods classified according to the NOVA system, and a qualitative component utilized focus group discussions, key informant interviews, and structured observations.

**Results:**

Galapagos residents increased consumption of processed and ultra-processed foods and decreased consumption of unprocessed and minimally processed foods. Perceived barriers to healthy diets include price, availability, and quality of fresh produce, as well as easy access to industrialized processed and ultra-processed foods.

**Conclusions:**

Changes in consumption patterns represent both local conditions and global trends; in that sense, the factors that affect Galapagos residents are not unique. Hence, these findings help elucidate processes observed in communities around the world.

## Background

Overweight and obesity represent a critical global public health problem; worldwide, the rate of obesity has tripled since 1975 [1]. As is the case in most countries, Ecuador is currently faced with the overweight and obesity pandemic, which affects all age groups beginning in early childhood [2]. Located 600 miles west of mainland Ecuador’s Pacific coast, Galapagos is best known for its unique natural environment, but it is currently home to 25,244 residents [3]. This figure contrasts with 1,350 residents in 1950; 9,785 in 1990; and 18,640 in 2001. Population growth was mainly a product of national migration, which is now regulated. On the other hand, while around 2,000 visitors toured the islands in the 1960s (mostly on boats), 224,745 registered tourists visited in 2015, many staying in hotels [4–6]. The challenges of balancing the benefits and pressures of tourism and conserving this World Heritage Site’s land and marine environment are daunting; moreover, local and national authorities must also attend to the health and nutrition of the islands’ residents [7]. While tourism is the major economic activity associated with the province, public administration and privately sector services are also important sources of employment. Recent regulations on the use of plastic reflects the importance of envorimental conservation and of the residents’ wellbeing [8]. Among Ecuador’s provinces, Galapagos has the highest rate of overweight and obesity in all age groups, including 12.7% among children under 5 and 75.9% among adults from 20 to 60. In comparision, the national average for adults is 65% and Latin American countries with the highest rates of adult overweight and obesity are Argentina (68%), Chile (65%), and Mexico (70%) [9, 10].

Galapagos may be viewed as different but not unique with respect to access, cost, and consumption of food, so an understanding of how those factors affect overweight and obesity can shed light on patterns observed in communities elsewhere in Ecuador and in other countries. In that sense, Galapagos represents a case study of the relationships between an emerging industrialized food regime and an increasingly prevalent local, regional, and global health problem [11–13]. For example, among nation states in the Pacific region, high rates of overweight and obesity have been shown to be related to consumption of imported, processed, and energy-dense foods instead of traditional diets, reflecting the emergence of complex, globalized commodity chains, urbanization, changes in occupational structures, and sedentary life styles, which characterize all countries to one degree or another [14–16]. Research on the nutritional transition shows that beginning in the 1970s, most countries experienced dietary shifts (especially, increased consumption of processed foods with high levels of fat, sugar, and salt and increases in away-from-home meals), and reduced physical activity. Consequently, rates of overweight and obesity and associated chronic diseases such as diabetes have increased worldwide. representing an emerging epidemic [17, 18]. This holds for Latin America, where obesity and overweight among children is of particular concern [19, 20].

The present study was designed to analyze overweight and obesity in Galapagos in the context of household food expenditures as well as perceptions and practices related to diet.

## Methods

The authors declare no conflicts of interest. This study was approved by the IRB of the Universidad San Francisco de Quito. It combined quantitative and qualitative research techniques in order to take advantage of the complementary strengths of each approach [21]. The quantitative component was based on the Ecuadorian Survey of Living Conditions (ECV, *Encuesta de Condiciones de Vida*)*,* which included 1,183 and 553 Galapagos households in 2009 and 2014, respectively, and which collected data on expenditures for food**s**, **which we** classified using the NOVA system [22, 23] based on a list of foods and beverages consumed in Ecuador [24]. ECV food expenditure data were matched with the Ecuadorian food composition tables [25], yielding a list of foods, categorized according to the NOVA system. The caloric content and the cost per calorie of each item were then calculated.

The NOVA system divides foods into four mutually-exclusive groups according to the level of processing. *Unprocessed or minimally processed foods* are unmodified or altered simply by separating edible from nonedible parts to preserve natural foods, enhance storability, or make foods safe to consume. *Processed culinary ingredients* contain oils, fat, sugar, or salt and are usually added to foods in the other groups. *Processed foods* are industrially transformed to increase the durability of unprocessed foods or to modify or enhance their sensory qualities. Examples include canned vegetables and fruits, cheese, and breads prepared by adding salt, oil, or sugar. *Ultra-processed foods* are manufactured products that include sweetened beverages, packaged snacks and desserts, reconstituted foods, meat products such as ham and sausages, and frozen or freeze-dried meals. These products include added sugar, fats, or salt as well as additional chemical or artificial ingredients, including colors or flavor enhancers [23].

The quantitative component of this study analyzed the contribution of foods in each of the four NOVA categories to the monthly household food basket in terms of calories and cost. Six demographic indicators were included in the analysis of household**s**: (i) urban or rural residence; (ii) the household member who was mainly responsible for purchasing food (PRP); (iii) age groups (20 to 39, 40 to 59, ≥ 60); (iv) level of formal education (none or primary, secondary, or university); (v) sex of PRP; and (vi) place of birth of the household head (Galapagos or other). The mean contribution value, 95% confidence interval, and a design-based sample t-test were calculated to evaluate significant differences between the two survey years for each categorical variable.

Poisson multivariable regression was used to evaluate the marginal effect of socioeconomic variables on the contribution of ultra-processed items to the food basket for each survey [26]. In order to compare both surveys, estimations were calculated for combined data with the year as a dummy variable. This step was possible because the sampling designs were similar for both surveys. Three different regression models were applied using data from: (i) 2009 and 2014 combined (Model A), (ii) 2009 (model B), and (iii) 2014 (model C). Statistical estimations of variance were calculated with Taylor series approximations, survey weights, and complex survey design using the R survey package [27].

Since households are not homogeneous, the statistical analysis began by normalizing expenditures and caloric content data in the four NOVA categories in order to compare average proportions in each household. This was accomplished by calculating the proportion of expenditures and calories in each category for each household and the average of proportions of expenditures and caloric content in each category. This information was disaggregated by urban or rural residence, age group, level of formal education, and sex. A T test was applied in order to establish differences between 2009 and 2014, disaggregated according to the different categories.

The study’s qualitative component was designed to understand perceptions and practices that shape patterns of food purchase and consumption among different segments of the Galapagos population [21, 28–30]. This component consisted of nine focus group discussions (FGD), 10 key informant interviews (KII), and structured observations (SO). FGD were conducted in the cities of Puerto Ayora (on the island of Santa Cruz) and Puerto Baquerizo (the provincial capital on the island of San Cristobal). In each city, four focus groups were conducted among adult women (≥ 20 years), adult men (≥ 20 years), unmarried male and female adolescents (12-19 years), and male and female children (8-11 years). An additional FGD was conducted with rural women on the island of San Cristobal. 38 (61.3%) of the participants were females and 24 (38.7%) were males. Perceptions of younger people were important to the study because of their influence in family food purchase and consumption, so 30 participants (48%) were between 8 and 11 years of age and 14 (11%) were between 12 and 17. All adult participants had attended at least six years of primary school; over a third attended or graduated from universities and an additional half were high school graduates.

KII were conducted with public health professionals, educators, and long-time residents of Galapagos in order to obtain information from persons with professional or personal knowledge about local dietary practices. SO were carried out in neighborhood shops, restaurants, municipal markets, and weekly farmers´ markets in order to assess food availability, quality, and cost of unprocessed or minimally processed, culinary, processed, and ultra-processed foods.

Focusing on how individuals perceive and navigate their social world, the qualitative component employed a theoretical sampling strategy to optimize validity by employing two operational principles. First, through triangulation, information was obtained from a variety of sources using a variety of techniques. Second, the principle of saturation was implemented: data collection continued until no additional information was obtained [31]. Verbatim transcriptions of FGDs and notes taken during KIIs and SO were analyzed in Spanish using a systematic three-stage coding process. First, open coding identified basic concepts enunciated by the participants in their own words. Second, axial coding provided for the development of underlying categories and properties in order to detect patterns of perceptions discussed by FGD participants. Third, selective coding integrated and refined the principal themes and interrelations among them to allow for the identification and analyisis of key dimensions [31].

## Results

### Quantitative component

Based on the NOVA classification, Table 1 presents mean percentages of monthly expenditures and mean percentage of calories purchased. Panel A shows a significant decrease in expenditures between 2009 and 2014 for non-processed and minimally processed foods and a significant increase in expenditures for processed and ultra-processed foods in urban areas, where about 90% of the population lives [3]. No differences are observed in rural areas. With regard to the mean proportion of calories purchased, a decrease is seen in the purchase of unprocessed foods in urban areas, while there is a significant increase of 8% in the purchase of ultra-processed foods. No significant differences are observed for rural residents.

**Table 1:** Mean monthly food expenditures and mean percentage of calories purchased

				Mean percentage of expended dollars NOVA food classification				Mean percentage of purchased calories NOVA food classification			
		Year	*n*	Unprocessed or Minimally processed	Processed culinary ingredient	Processed	Ultra-processed	Unprocessed or minimally processed	Processed culinary ingredient	Processed	Ultra-processed
A. Area	Urban	2009	931	73.2 (72.3-74.1)***	5.4 (5.1-5.6)*	7.4 (7.0-7.8)***	16.8 (15.8-17.9)	58.3 (56.8-59.8)***	17.7 (17.0-18.5)	5.9 (5.4-6.4)	21.3 (19.6-23.0)***
	Urban	2014	486	69.2 (67.7-70.8)	6.1 (5.6-6.6)	11.2 (9.9-12.6)	19.3 (17.0-21.6)	52.3 (49.8-54.9)	18.7 (17.1-20.4)	5.4 (4.9-5.8)	30.3 (26.7-34.0)
	Rural	2009	306	76.4 (75.2-77.5)	5.9 (5.3-6.5)	7.3 (6.8-7.8).	11.7 (10.5-12.9)	67.8 (65.6-70.0)	16.4 (15.0-17.8)	6.0 (5.4-6.7)	10.9 (9.0-12.8).
	Rural	2014	82	74.0 (71.2-76.7)	5.6 (5.1-6.0)	8.6 (7.3-10.0)	14.8 (11.2-18.4)	62.5 (55.4-69.7)	17.5 (15.8-19.1)	5.5 (5.0-6.0)	19.2 (11.1-27.2)
B. Age in years	>60	2009	183	74.9 (72.1-77.6)*	5.9 (5.2-6.5)*	7.4 (6.5-8.2)**	16.8 (13.1-20.6)	61.6 (57.9-65.3)*	18.1 (16.3-20.0).	5.8 (4.8-6.8)	20.6 (16.0-25.3)
	>60	2014	78	69.6 (65.7-73.5)	7.4 (6.1-8.6)	10.7 (8.7-12.8)	16.5 (12.5-20.5)	54.8 (49.6-60.0)	22.0 (18.5-25.5)	5.5 (4.4-6.7)	23.2 (17.8-28.6)
	40-59	2009	589	74.2 (73.2-75.3)	5.5 (5.1-5.8)	7.6 (7.0-8.2)**	14.6 (13.5-15.7)	61.5 (59.8-63.2)**	17.5 (16.6-18.4)	6.1 (5.5-6.7).	17.2 (15.4-19.0)***
	40-59	2014	277	72.6 (70.9-74.3)	5.6 (5.1-6.0)	9.9 (8.6-11.1)	16.4 (14.1-18.7)	55.6 (52.2-58.9)	17.7 (16.2-19.3)	5.3 (4.8-5.9)	26.9 (22.6-31.2)
	20-39	2009	463	72.4 (71.1-73.7)**	5.3 (5.0-5.6)*	7.0 (6.6-7.5)***	18.0 (16.5-19.5)	56.3 (53.8-58.8)	17.4 (16.2-18.5)	5.6 (4.9-6.4)	23.3 (20.6-26.1)**
	20-39	2014	212	67.6 (64.6-70.5)	6.0 (5.4-6.5)	11.6 (9.5-13.6)	21.6 (17.8-25.4)	53.6 (50.0-57.3)	17.9 (16.4-19.4)	5.4 (4.9-6.0)	31.0 (26.2-35.7)
C. Education	None or Primary	2009	400	76.4 (75.1-77.7)**	6.0 (5.6-6.4)*	7.0 (6.6-7.5)***	13.0 (11.3-14.7)	65.6 (63.1-68.1)**	16.8 (15.6-18.1)*	5.1 (4.6-5.6)	15.5 (12.8-18.3)**
	None or Primary	2014	126	72.4 (69.7-75.0)	7.1 (6.2-8.0)	10.7 (8.9-12.4)	16.2 (12.5-19.9)	57.2 (51.6-62.8)	19.8 (17.4-22.3)	5.2 (4.4-6.0)	25.0 (19.2-30.8)
	Secondary	2009	571	73.0 (71.8-74.1)**	5.2 (4.9-5.5).	7.5 (6.9-8.1)***	17.0 (15.7-18.2)	57.0 (55.0-59.0)	17.7 (16.8-18.7)	6.2 (5.4-7.0).	21.4 (19.2-23.6)**
	Secondary	2014	276	69.7 (67.7-71.7)	5.8 (5.3-6.3)	11.3 (9.6-12.9)	19.9 (17.0-22.8)	54.8 (51.5-58.0)	18.1 (16.5-19.7)	5.2 (4.7-5.8)	29.8 (25.3-34.4)
	Univ or Higher	2009	266	71.4 (69.5-73.3)	5.2 (4.7-5.7)	7.5 (6.8-8.2)*	18.6 (16.6-20.6)	57.1 (54.5-59.7)*	18.1 (16.6-19.6)	6.4 (5.6-7.1)	22.5 (19.6-25.4).
	Univ or Higher	2014	166	69.8 (67.2-72.3)	5.3 (4.8-5.9)	9.5 (8.1-10.8)	17.2 (15.0-19.4)	52.7 (49.5-56.0)	17.8 (16.1-19.6)	5.8 (5.2-6.5)	26.7 (22.8-30.6)
D. Sex	Male	2009	419	72.0 (70.2-73.8)**	5.4 (5.0-5.8)*	8.1 (7.1-9.1)***	20.0 (17.9-22.1)*	54.4 (51.6-57.2)*	17.7 (16.5-18.9)	6.8 (5.8-7.8)	26.5 (23.3-29.7)**
	Male	2014	156	65.8 (62.4-69.3)	6.6 (5.7-7.4)	14.5 (11.6-17.3)	25.0 (20.6-29.4)	48.3 (43.7-52.9)	19.9 (17.2-22.5)	6.3 (5.4-7.1)	38.8 (32.3-45.3)
	Female	2009	818	74.3 (73.5-75.2)**	5.5 (5.2-5.7)	7.0 (6.7-7.4)***	14.4 (13.5-15.2)	61.9 (60.5-63.4)**	17.5 (16.7-18.3)	5.5 (5.1-6.0)	16.9 (15.4-18.4)***
	Female	2014	412	71.9 (70.3-73.5)	5.8 (5.4-6.2)	9.3 (8.4-10.1)	15.8 (13.8-17.8)	57.0 (53.9-60.1)	18.0 (16.6-19.4)	5.1 (4.7-5.4)	23.8 (20.3-27.4)
E. Total	Total	2009	1237	73.6 (72.8-74.4)***	5.5 (5.2-5.7)*	7.4 (7.0-7.7)***	16.2 (15.3-17.1).	59.6 (58.2-60.9)**	17.5 (16.9-18.2)	5.9 (5.5-6.3).	20.0 (18.5-21.4)***
	Total	2014	568	70.4 (68.9-71.9)	6.0 (5.6-6.4)	10.6 (9.5-11.7)	18.3 (16.2-20.4)	54.8 (51.7-57.8)	18.4 (17.1-19.7)	5.4 (5.0-5.7)	27.9 (24.1-31.6)

Panel B shows that among people from 20 to 39 years and 60 years or more, the mean proportion of expenditures for unprocessed and minimally-processed foods significantly decreased, while in all age groups, the mean proportion of expenditures for processed foods increased. Similarly, a reduction is observed in calories purchased in unprocessed foods among persons 40 years of age and more, while the calories purchased in ultra-processed foods increased significantly in people 59 years of age or more.

Panel C shows that the proportion of mean expenditures for unprocessed and minimally processed foods declined between 2009 and 2014 among persons with a high school education or less, while the proportions increased in the same group for processed and ultra-processed foods. Observing the mean proportion of calories purchased, a significant decrease in unprocessed or minimally processed foods can be seen in persons with a primary education or less and those with a college education, but not among those with a secondary education. But the mean proportion of calories purchased for ultra-processed food increased in persons with secondary education or less.

Panel D shows that mean proportions of expenditures for unprocessed and minimally processed foods declined between 2009 and 2014 among men and women who were responsible of food purchase, while expenditures for processed foods increased among men and women. But the expenditures for ultra-processed foods were significantly higher among men than women. In observing mean proportions of calories purchased, a significant decrease is seen in unprocessed and minimally processed foods among men and women, but there was a significant increase in ultra-processed foods.

Finally, Panel E shows that mean proportions of expenditures for unprocessed and minimally processed foods decreased significantly between 2009 and 2014, while expenditures for ultra-processed and processed foods increased (the latter, significantly). In terms of calories purchased, a significant decrease is seen in unprocessed and minimally processed foods, along with an increase in calories purchased in processed foods and ultra-processed foods (the latter difference is significant).

As presented in Table 2, Poisson multivariable regressions show that when males were responsible for purchasing food, the contribution of ultra-processed items to the food basket increased by 0.2% (95% CI: 0.1, 0.3). Similarly, years of education is significant; respondents with more education spent more on ultra-processed foods than their less educated counterparts (0.2%; 95% CI: 0.1, 0.4), while rural or urban residence is only significant for 2009. Expenditures for ultra-processed foods were higher among urban than rural residents; the overall model increased for urban residency by 0.3% (95% CI: 0.1, 0.4). When adjusting for other co-variables, the effect of age was not significant in the regression analysis. Finally, the analysis of expenditures shows that the cost per calorie of ultra-processed foods was significantly higher than for non-processed foods, and that when the cost of non-processed foods increases, there is a corresponding increase in the cost of ultra-processed foods.

**Table 2:** Relationship between relative ultra-processed food expenditure and demographic determinants (2009-2014, 2009, 2014)

	Model A (2009-2014)	Model B (2009)	Model C (2014)
pNPc^1^	-0.5*** (-0.5, -0.4)	-0.5*** (-0.6, -0.4)	-0.5*** (-0.5, -0.4)
pUPc^2^	0.2*** (0.2, 0.3)	0.2*** (0.2, 0.3)	0.2*** (0.2, 0.2)
Sex: Male	0.2*** (0.1, 0.3)	0.2*** (0.1, 0.3)	0.2** (0.02, 0.3)
Education: Secondary	0.1** (0.01, 0.3)	0.2** (0.01, 0.3)	0.1 (-0.05, 0.3)
Education: Superior	0.2*** (0.1, 0.4)	0.2*** (0.1, 0.4)	0.2** (0.03, 0.3)
Age: 20-39 years	0.1 (-0.1, 0.3)	0.1 (-0.1, 0.3)	0.1 (-0.2, 0.4)
Age: 40-59 years	0.03 (-0.2, 0.2)	0.04 (-0.1, 0.2)	0.03 (-0.2, 0.2)
Area: urban	0.3*** (0.1, 0.4)	0.3*** (0.2, 0.5)	0.1 (-0.2, 0.4)
Year2014:pUPc2	-0.1*** (-0.1, -0.01)		
Constant	0.7*** (0.2, 1.2)	0.4 (-0.2, 1.0)	0.8** (0.2, 1.5)
Observations	1.736	1.183	553
Log Likelihood	-512,618	-407.44	-253,853
Akaike Inf. Crit.	1,045.235	832,879	525,706

^1^Log price of mean non processed and minimally processed calorie (USD/Cal)

^2^Log price of mean ultra-processed calorie (USD/Cal)

*p<0.1; **p<0.05; ***p<0.01

### Qualitative component

Three principal dimensions emerged from the qualitative data analysis: dietary quality, characteristics of the daily diet, and factors that influence the purchase of food for home consumption.

#### Dietary quality

When asked to describe healthy diets in general, most respondents discussed their own diets as unhealthy and unbalanced because of bad habits, dependence on restaurant meals, and large serving sizes. (See Table 3, panel A.) The content of food was also perceived to contribute to unhealthy diets; participants explained that their diets are unbalanced because they lack variety, being dominated by white rice, to which are added pasta, potatoes, or fried plantains, accompanied by portions of fried meat. While participants stated that they consume fruit (either whole or in blended drinks made with sugar), consumption is not frequent. Participants also recognized that their meals are unhealthy because food is usually fried in oil, and even soups (a nearly ubiquitous component of lunch menus) can be fat-laden. One KI, a hospital director, observed that Galapagos residents eat surprisingly little fish, while another physician added that poorer residents in particular have poor diets.

**Table 3:** Perceptions related to diet and overweight and obesity in Galapagos

A. Dietary quality	
*A lot of sugar; a lot of salt; a lot is fried. Everything is unhealthy these days. (Adult female)*	
*(In the school cafeteria), la food isn’t completely health as it should be. There is food with fat, those sorts of things. Also, I like sweets and I always eat them. So it’s not as health a diet as it should be. (Adolescent 16-18, female).*	
*I think that what my mother gives me is health because she gives me vegetables and all those things, like soup. (Boy, 8-12)*	
*It’s just that rice should never be lacking in the house, I think. . . . Pasta with rice, fried pork with rice, corn. (Adult female)*	
*The soups have a lot of fat, and the rice also has some more fat, and no restaurant that we’ve gone to serves a salad. (Adult female)*	
*There was rice there; I reheated some rice and I bought a pack of four hot dogs: big ones, I’d say. Without thinking—I don’t know how to economize—I took them and I said, today I’m going to eat well. So four hot dogs: two for (my daughter) and two for me. (Adult female)*	
*I really don’t know much about what a healthy diet is. . . . I try to vary (the food) but exactly how to do that, I have no idea. (Adult female)*	
*One knows what to do, but not how to eat [healthy]. (Adult male)*	
*During this year that I´ve been able to be at home, I’ve been trying to go back to natural [foods] and trying to get rid of [unhealthy food]. (Adult female)*	
*For example, when they bring tomatoes from the highlands, they come in trucks. How do they come? How many days does it take those trucks to get to the dock, which is the shipping point in Guayaquil, and until it is loaded? (Adult female)*	
B. Daily diet	
*I don’t buy snacks in the cafeteria. When I leave school, I buy little pastries that cost 25 cents and in the middle of the afternoon, I buy a fried plantain ball or ice cream. (Boy, 8-12 years)*	
*My diet is healthy because even though I eat junk food, which you can understand from the labels, like potato chips. I also practice sports and I burn calories. (Girl, 8-12 years)*	
C. Factors in purchasing food for home consumption	
*Recently I started growing tomatoes, green peppers, and things like that. (Adult female)*	
*I have two papaya trees in my yard, so when my children want papaya, I can use those. (Adult female)*	
*We can´t grow much in the highlands. We have only family agriculture because the worms eat everything, and it’s dry. We can’t use fungicides or insecticides because they are prohibited in Galapagos. But the water is in heavy demand; worse now in the dry season, and it’s almost impossible to carry water (for) the plants. (Adult female)*	
*In reality, we know that we should eat vegetables and fruit, but you could say that it’s a luxury.* (*Adult female)*	
*Many people here don’t have a balanced diet because of the cost and salaries. One cannot buy what one wants because food goes through a process in order to arrive here, so the price increases. To buy fruit, it costs you double what it costs elsewhere and sometimes it is not within peoples’ budgets.* (*Adolescent, 12-17****,*** *female)*	
*If you need something specific, you have to look for it throughout the island, and sometimes you can’t find it anywhere (Adolescent, 12-17, female).*	
*(When buying produce, you have to look at) the price, expiration date, and if it is clean. (Girl, 8-12).*	
*My mother and father (buy food); I always stay home When I do go, I get my cookies or something like that, but I have to look at the price, in the nutritional label, the quality, and when it expires because sometimes food stays on the shelf and if we don’t use it right away, we have to throw it away (Girl, 8-12)*	
*There are times that something happens to the ship and you don’t find (produce) for a week or two weeks. (Adult female)*	
*It’s also how the vegetables get here. For example, the plantains come in those containers and also other fruit. But when they open (the containers) the rats and all those animals come out, and that’s what we eat. (Adult female)*	
*It’s enough that there is red. For example, I taught this to my daughter because when we shop, she always wants to grab something without looking and I say that there is red, so no. (Adult female)*	
*I see that it’s high in sugar if it’s red. (Girl 8-12)*	
*We bought a big package last Christmas and we only finished it two weeks ago. (Adult male)*	
*I look at the labels of processed meat and we don’t buy very much. (Adult male)*	

These points are critical because many participants reported that they frequently eat lunch in restaurants, where consumers exerecise little control over the content of their food. Additionally, many families regularly eat out on weekends. Another factor related to unhealthy diets is large portion sizes served at home and in restaurants, where serving size is not controlled. Adult males and females FGD participants largely agreed on this point; while women purchase most food, some families shop together on weekends. Children and adolescents placed most of the responsibility for their diets on their parents, noting that they sometimes are given money to buy snacks.

The relationship between knowledge and practice also affects food consumption. While some participants felt that they don’t know enough about healthy diets or how to translate knowledge into practices to control diet, others felt that they do know, having obtained information from health professionals or the internet. In some cases, participants stated that they have changed their diets by incorporating healthier foods. Others acknowledged that they know about healthy diets but do not necessarily purchase or consume healthy foods. Men, women, adolescents, and even children reported that the most significant obstacle to bridging the gap between knowledge and practice is the high or even prohibitive cost of fresh produce--approximately twice as much as in mainland Ecuador. By the time fresh produce is sold locally, it is also often in poor condition, having spent days or weeks in transit. A related factor is the variation in availability due to seasonality and difficulties in acquiring and distributing fresh products on a regular basis. Additionally, much of the fresh produce is delivered to restaurants and the tourism industry rather than to consumers.

#### Daily diet

When asked to describe what they ate the day before the FGD, participants were remarkably uniform in their responses. (See Table 3, Panel B.) Breakfast is normally a light meal of bread or toast, fruit juice prepared with sugar, coffee or tea, and sometimes, yogurt or eggs. In contrast, lunch is the heaviest meal, whether consumed at home, in a restaurant, or in a school cafeteria. It usually starts with a carbohydrate-rich soup prepared with wheat or barley flour, potatoes, or pasta; followed by a main dish consisting of fried meat served with two to three cups of cooked white rice, a small salad, and a dessert prepared with sugar. Students lunch at home after classes or in school cafeterias, restaurants, or street kiosks. School menus often feature processed and ultra-processed foods; sweetened carbonated beverages are not sold, but fruit juices prepared with sugar are.

Male and female FGD participants report that dinner is usually a lighter meal of a sandwich or toast, eggs, or fried plantains. Participants also reported frequent between-meal snacks that often include baked goods (bread, cookies, cake, or cupcakes), sandwiches, or chips. Water may be consumed, as are sweetened beverages, including carbonated drinks, juices, or bottled ice tea. Notably, while some respondents avoid snacking, others, especially women who work at home, report that they snack constantly, while students reported consuming ultra-processed foods such as chips during recess. Schoolchildren also report that they buy snacks such as ice cream or pastries on the way to or from school. Some childen and adolescents understand that their snacks are not healthy, but justify them by asserting that consumption is compensated for by physical activity. Nevertheless, respondents of all age groups reported limited activity (often concentrated in a few hours on weekends); a KI physician and an educator commented that physical activity during school recess is often absent or not very vigorous.

#### Factors in purchasing food for home consumption

FGD participants identified four issues related to opportunities and barriers to purchasing healthy food for home preparation and consumption. (See Table 3, Panel C.) The most important was place of purchase; FGD particpants and four KIs reported that virtually all consumers purchase food in small neighborhood shops, which offer a limited variety of goods. Processed and ultra-processed foods share space with household supplies, and most shops have limited refrigerated space that is mostly dedicated to milk, milk products, and other beverages. Limited quantities of fresh produce are found on shelves, in boxes on floors, or in a few cases, in refrigerators.

Alternatives to neighborhood shops include municipal markets, which offer a limited range of produce that is either locally sourced or brought from the mainland. There is only one such market in Puerto Ayora and one in Puerto Baquerizo, however. Additionally, a few specialized shops offer meat and seafood, some of which is locally sourced, and weekly farmers’ markets have been organized in both cities in order to link consumers with local producers. While some respondents prefer farmers’ markets, others stated that they were not aware of their existence. Some households have direct access to fresh food, either because they have small farms or gardens in their yards. FGD participants—especially women who purchase food--and KIs agreed that they prefer fruit and vegetables from the mainland because they are larger and in better condition than local produce. Moreover, local production is limited because of persistent drought, insufficient irrigation water, insect infestation, and regulations that limit or prohibit the use of agrochemicals.

A second factor is frequency of food purchase. As is the case elsewhere in Ecuador, consumers purchase some items every day, while other items are purchased on a weekly basis, including fresh produce in neighborhood shops, municipal markets, or farmers’ markets. Purchase of these items is conditioned by availability, which is irregular due to the vicissitudes of transportation from the mainland and the variable ability of shops to acquire those products.

Third, male and female adults base food purchase decisions on price and quality, and even children and adolescents were sensitive to those factors. In particular, the cost of fresh produce limits consumption, even when nutritional advantages are recognized. FGD participants reported that they assess quality in terms of freshness and the appearance of fruit and vegetables. In this sense, the preference for processed and ultra-processed foods is based on perceived lower comparative cost and because quality is standardized and does not deteriorate. Respondents stated that brand preferences are not important because options are limited, while expiration dates are important because of the time it may take for packaged foods to reach them.

Fourth, FGD and KII participants expressed different perceptions about the Ecuadorian traffic light nutritional food label. Even children and adolescents reported that they notice the label, although adolescents are less likely to change consumption decisions because of the information on the label. Their responses coincide with findings reported in a recent national qualitative study [22] in that the label is recognized and understood because it is simple and colorful: red, yellow, and green are associated with high, medium, and low levels of added fat, sugar, and salt. While the label was designed to provide easily-understood information, the use to which respondents put it varies. Some reported compensating for what they regard as an unhealthy diet by exercising, adding less sugar to beverages, purchasing processed or ultra-processed foods less frequently, or by consuming smaller quantities than previously.

## Discussion

The prevalence of obesity and overweight in Galapagos is higher than elsewhere in Ecuador [2], affecting residents in all age groups. Rates are also high compared to other countries, and as elsewhere, increasing consumption of processed and ultra-processed foods influences dramatic and troubling trends in the health and nutrition profile in Galapagos [32]. Galapagos residents face substantial barriers to the purchase and consumption of healthy foods (especially fresh fruit and vegetables). At the same time, they spend increasingly less on unprocessed and minimally processed foods and more on processed and ultra-processed foods and proportionally more on calories associated with the latter as compared to the former. A pattern of disparity with regard to the selection of unhealthy foods is observed, in particular, when males purchase food, among less educated persons, and among urban residents. While issues of cost clearly are in the forefront in this regard, this pattern also suggests that programs of promotion and prevention could be oriented toward specific groups in schools, shops, restaurants, health centers, and in mass media.

The qualitative component of this study suggests that prices for fresh produce are considered to be prohibitively high, availability is irregular, quality is often poor, and access to locally produced food is limited. This component confirms that expenditures are increasingly dedicated to the purchase of processed and ultra-processed foods, thereby resulting in a shift in food consumption patterns. While this trend characterizes all families to some degree, expenditures for processed and ultra-processed foods are greatest in urban households where males are responsible for purchasing food and when educational levels are higher.

Overweight and obesity respresent a challenge to public health in Galapagos, while food expenditures and consumption patterns reflect cost advantages of processed and ultra-processed foods and more broadly, the globalization of an increasingly industrialized food chain [33, 34]. Broad socioeconomic transformations that affect Galapagos (notably, urbanization and sedentary occupations and lifestyles) are important factors. Measures that should be taken in Galapagos may be valid elsewhere, although implementation strategies will necessarily vary. First, the availability and quality of fresh foods should be improved and local production of fruit and vegetables should be incentivized in order to improve dietary quality and reduce dependence on imported foods [35]. Second, a multi-faceted nutritional education program should include easily-understood information provided to parents and children. Third, policy options could include maintaining the current nutritional label and regulating or taxing sweetened beverages. Third, family, community, and school gardens should be promoted by the Ministries of Education and Agriculture, and incentives provided to local small-scale farmers. Agricultural production and urban residence represent only 3% of total land area in Galapagos [36], but the need for policies that are consistent with conservation is clear. Such a program should be established within the paraters set by the Galapagos National Park, which sets guidelines for all non-urban land use, and the Galapagos Biosecurity Agency, which regulates the control of insects and prohibit most pesticide use. It would concentrate on sustainable use of existing agricultural land and urban gardens, efficient use of water, organic production and pest control techniques, and enhanced linkages between Galapagos producers and consumers. This approach would be consistent with conserving the unique natural resources of the Galapagos.

Finally, mass media and other communication channels should provide information about healthy diets, and restaurants should receive incentives for providing nutritional information and for serving healthy foods.

## Conclusion

While the residents of Galapagos face specific constraints to healthy dietary practices, these constaints are not unique but are, rather, accentuated due to the islands’ characteristics—especially their relative isolation and vulnerability. Access to healthy diets are limited to one degree or another in communities and countries worldwide, while at the same time, globalized and industrialized processed and ultra-processed foods increasingly contribute to the worldwide pandemic of overweight and obesity [37]. Local food consumption is shaped by both the tourist industry (because of the competing demands for high-quality fresh food and produce) and regulations on land use, but all local populations face limitations and opportunities for healthy consumption, and in this sense, Galapagos is, indeed, a window on the world.

This study benefitted from the intersection of quantitative methods, which allowed for analyzing statistical differences in food expenditures, and qualitative methods, which in themselves are not necessarily generalizable, but which are contextualize quantitavie findings in terms of the perceptions and practice of Galapagos residents. Qualitative methods alone do not allow for extrapolation, but they are invaluable in elucidating relationships established using quantitative techniques [28–30, 34]. The NOVA classification system [22, 23] provides a useful way to analyze patterns and changes in food purchase and consumption among households with different demographic characteristics.

## Abbreviations

ECV: Survey of Living Conditions
FGD: Focus group discussions
KII: Key informant interviews
PRP: Responsible for purchasing food
SO: Structured observations
